# Survey and improvement strategies for gene prioritization with large language models

**DOI:** 10.1093/bioadv/vbaf148

**Published:** 2025-06-24

**Authors:** Matthew B Neeley, Guantong Qi, Guanchu Wang, Ruixiang Tang, Dongxue Mao, Chaozhong Liu, Sasidhar Pasupuleti, Bo Yuan, Fan Xia, Pengfei Liu, Zhandong Liu, Xia Hu

**Affiliations:** Jan and Dan Duncan Neurological Research Institute at Texas Children’s Hospital, Houston, TX, 77030, United States; Graduate School of Biomedical Sciences, Program in Quantitative and Computational Biosciences, Baylor College of Medicine, Houston, TX, 77030, United States; Jan and Dan Duncan Neurological Research Institute at Texas Children’s Hospital, Houston, TX, 77030, United States; Graduate School of Biomedical Sciences, Program in Genetics and Genomics, Baylor College of Medicine, Houston, TX, 77030, United States; Department of Computer Science, Rice University, Houston, TX, 77005, United States; Department of Computer Science, Rutgers University, New Brunswick, NJ, 08854, United States; Jan and Dan Duncan Neurological Research Institute at Texas Children’s Hospital, Houston, TX, 77030, United States; Department of Pediatrics, Baylor College of Medicine, Houston, TX, 77030, United States; Department of Molecular and Human Genetics, Baylor College of Medicine, Houston, TX, 77030, United States; Jan and Dan Duncan Neurological Research Institute at Texas Children’s Hospital, Houston, TX, 77030, United States; Graduate School of Biomedical Sciences, Program in Quantitative and Computational Biosciences, Baylor College of Medicine, Houston, TX, 77030, United States; Jan and Dan Duncan Neurological Research Institute at Texas Children’s Hospital, Houston, TX, 77030, United States; Department of Pediatrics, Baylor College of Medicine, Houston, TX, 77030, United States; Department of Molecular and Human Genetics, Baylor College of Medicine, Houston, TX, 77030, United States; Human Genome Sequencing Center, Baylor College of Medicine, Houston, TX, 77030, United States; Department of Molecular and Human Genetics, Baylor College of Medicine, Houston, TX, 77030, United States; Baylor Genetics, Houston, TX, 77021, United States; Department of Molecular and Human Genetics, Baylor College of Medicine, Houston, TX, 77030, United States; Baylor Genetics, Houston, TX, 77021, United States; Jan and Dan Duncan Neurological Research Institute at Texas Children’s Hospital, Houston, TX, 77030, United States; Department of Pediatrics, Baylor College of Medicine, Houston, TX, 77030, United States; Department of Computer Science, Rice University, Houston, TX, 77005, United States

## Abstract

**Motivation:**

Rare diseases remain difficult to diagnose due to limited patient data and genetic diversity, with many cases remaining undiagnosed despite advances in variant prioritization tools. While large language models have shown promise in medical applications, their optimal application for trustworthy and accurate gene prioritization downstream of modern prioritization tools has not been systematically evaluated.

**Results:**

We benchmarked various language models for gene prioritization using multi-agent and Human Phenotype Ontology classification approaches to categorize patient cases by phenotype-based solvability levels. To address language model limitations in ranking large gene sets, we implemented a divide-and-conquer strategy with mini-batching and token limiting for improved efficiency. GPT-4 outperformed other language models across all patient datasets, demonstrating superior accuracy in ranking causal genes. Multi-agent and Human Phenotype Ontology classification approaches effectively distinguished between confidently-solved and challenging cases. However, we observed bias toward well-studied genes and input order sensitivity as notable language model limitations. Our divide-and-conquer strategy enhanced accuracy, overcoming positional and gene frequency biases in literature. This framework optimized the overall process for identifying disease-causal genes compared to baseline evaluation, better enabling targeted diagnostic and therapeutic interventions and streamlining diagnosis of rare genetic disorders.

**Availability and implementation:**

Software and additional material is available at: https://github.com/LiuzLab/GPT-Diagnosis

## 1 Introduction

Rare diseases affect a small fraction of the population but collectively impose a significant burden upon individuals and society (Robinson *et al.* 2020). These rare conditions are frequently of genetic origin and classified as Mendelian diseases and result from mutations in single genes. Their rarity and diversity considerably complicate diagnosis and treatment. A notable portion of these conditions remains undiagnosed due to the unpredictability and complexity of their genetic foundations and clinical presentations, leading to lifetimes of difficulty and uncertainty for patients and their families (Marwaha *et al.* 2022). There is an urgent need for enhanced diagnostic tools and methodologies to evaluate patient data.

In response to this need, clinical sequencing, including whole genome sequencing (WGS) and exome sequencing (ES), has become an indispensable tool in the exploration of rare diseases. These techniques allow clinicians and researchers to meticulously analyze a patient’s genome and detect potentially causative genetic variants. While the vast array of candidate variants presents a challenge, variant prioritization tools effectively address this issue.

Utilizing gene prioritization to reliably identify the causal disrupted gene is critical for accurate diagnosis and understanding the genetic variants responsible for the disease (Stelzer *et al.* 2016, Rao *et al.* 2018). Other approaches to gene prioritization depended largely on structured databases that compile well-known genetic data and predictions regarding the impact of various mutations often coupled with traditional machine learning methodologies (Jacobsen *et al.* 2022, Yuan *et al.* 2022).

Innovative solutions in artificial intelligence have been primarily driven by the development of LLMs like GPT-4, Llama2 (Touvron *et al.* 2023), and Mixtral-8x7b (Jiang *et al.* 2024). These models are trained on a wide array of massive data sources, including textual datasets, programming code bases, and other forms of digital content. Such training enables these models to not only emulate human-like communication but also to acquire a substantial understanding of specialized domains, including medical sciences (Singhal *et al.* 2023, Yuan *et al.* 2023, Mehandru *et al.* 2024, Perlis *et al.* 2024). Unlike simple data repositories, LLMs exhibit advanced reasoning capabilities, allowing them to process and interpret extensive and unstructured datasets effectively. This attribute is particularly valuable in exploring underutilized applications in genetic research and the diagnosis of rare diseases (Vaswani *et al.* 2017).

LLMs have been shown to excel at and achieve state-of-the-art performance in a variety of tasks and benchmarks (Wang *et al.* 2020, Bubeck *et al.* 2023, Yuan *et al.* 2023). Recently, in the medical setting, researchers found that LLMs outperform medical experts at summarizing electronic health records (Van Veen *et al.* 2024). Recent studies highlight the promise of LLMs for gene prioritization in rare diseases. Kim *et al.* (2024) demonstrated phenotype-driven prioritization passing the phenotype descriptions without genetic information to LLMs and nominating candidate causal genes, noting biases toward frequently studied genes. Flaharty *et al.* (2024) explored LLM performance in syndrome recognition from diverse phenotypic descriptions. Additionally, Toufiq *et al.* (2023) applied LLMs to prioritize genes within specific co-expression modules, evaluating their relevance across multiple biological dimensions. These advances underscore LLM versatility in addressing rare-disease diagnostic challenges.

In the context of rare disease, LLMs offer significant advantages, in which traditional data sources are scarce and unique (Marwaha *et al.* 2022). LLMs excel at parsing structured and unstructured data and at converting clinical narratives and genetic information into structured data that enhances gene-based prioritization. Unlike traditional machine learning methods that require large datasets for training, LLMs can make a diagnosis prediction from a single case without task specific pre-training (Brown *et al.* 2020, Bubeck *et al.* 2023). This capability allows LLMs to provide valuable insights and predictions even when working with limited and unique patient cases. Two key scenarios illustrate the utility of LLMs in gene prioritization: In the Undiagnosed Diseases Network (UDN), where the diagnostic process depends on allele frequency, phenotype matching, and expert input, LLMs can simulate expert panels, quickly accessing curated literature and data to prioritize candidate genes. In routine diagnostic practice, clinical geneticists use AI tools like AI MARRVEL (AIM) (Mao *et al.* 2024) and Exomiser (Smedley *et al.* 2015) to narrow down genetic variant lists; our study mirrors this by implementing a 50-gene ranking task to assist geneticists in focusing on the most likely disease-causing candidates. These examples show that LLMs enhance molecular diagnosis by prioritizing genes based on phenotype relevance, literature support, and expert knowledge, addressing real-world clinical challenges.

In comparison to traditional methods for gene prioritization, LLMs have the potential to offer new insights into the diagnosis of rare diseases by providing a more accurate and interpretable measurement of phenotype–genotype relationships. Our preliminary analysis showed LLMs performed as well or better than traditional methods when measuring phenotype–genotype relationship, such as phrank score (Jagadeesh *et al.* 2019) (Supplementary Fig. S1, available as supplementary data at *Bioinformatics Advances* online). Additionally, our LLM-based approach improved the ranking of true disease-causing genes in 49 of 145 patient cases for which AIM, the state-of-art variant prioritization method failed to identify the disease-causal variants. Furthermore, our study also serves as a critical baseline, offering valuable direction for future improvements in AI methodologies for clinical applications such as incorporating LLMs downstream of AIM in cases that were unsolved to recover a portion of those cases.

In this study, we explore the potential of LLMs to enhance gene prioritization by effectively analyzing clinical phenotypes and candidate disease-causing genes to identify the diagnostic gene. Utilizing LLMs allows us to refine both the accuracy of gene-based prioritization and the process by which we diagnose rare genetic disorders, ultimately leading to more precise and informed medical interventions.

## 2 Methods

### 2.1 Dataset

Because there is not currently a consensus strategy for benchmarking gene-based prioritization methodology, synthetic datasets are often selected for evaluation (Guala and Sonnhammer 2017). We, however, used a large dataset of resolved patient cases: 1063 cases from a clinical diagnostic lab, Baylor Genetics (BG); 90 from the Undiagnosed Diseases Network (UDN); and 200 from Deciphering Developmental Disorders (DDD). The patient data consists of phenotypes from clinical electronic health records (EHR) and genomic sequencing data.

We preliminarily filtered raw genomic data from each patient to keep variants with an allele frequency less than 1%, or that were classified as pathogenic or likely pathogenic in ClinVar or HGMD, or that had a SpliceAI score greater than 0.8. The variants were converted to the gene level for gene-based prioritization. We then generated three gene sets of different sizes (5, 25, and 50 genes) for each patient by randomly sampling from their noncausal genes and adding the causal gene. We shuffled the genes to randomize to the gene ordering for the prompt. To determine the dataset sizes (5, 25, and 50 genes), we simulated real-world scenarios where geneticists or expert panels prioritize genes based on phenotype relevance and available data. The 5-gene set reflects a focused, expert-driven analysis, as seen in the UDN, while the 25- and 50-gene sets mirror broader clinical geneticist practices that involve larger pools of candidate genes. These different sizes impact diagnosis accuracy by testing how well LLMs can prioritize the correct gene as the pool of candidates increases, with the accuracy expected to decline as the number of genes expands due to the complexity of narrowing down to a single correct diagnosis. We finally converted patient clinical phenotypes from numerical HPO terms to their corresponding definitions because LLMs tend to hallucinate the true meaning of HPO terms (Supplementary Fig. S13, available as supplementary data at *Bioinformatics Advances* online).

### 2.2 Prompt creation

We enumerated clinical phenotypes and candidate genes to structure the prompt. Then, we included instructions for the model to assign a rank based on association between the specified genes and the clinical phenotypes. We included further guidance regarding the use of gene function, expression sites, or analogous animal model information in the absence of direct empirical data. As a final point, to precisely define the response format, we instructed the model to rank gene associations by probability (0–1.0), emphasizing brevity and the exclusion of explanatory justifications (Supplementary Fig. S2A, available as supplementary data at *Bioinformatics Advances* online).

### 2.3 Multi-agent classification

The Multi-Agent Classification method is an approach we developed for classifying patients by phenotypes via a multi-agent LLM pipeline that consists of two main steps (Supplementary Fig. S7, available as supplementary data at *Bioinformatics Advances* online). In the first step, the original prompts are given to an evaluator agent (GPT-4) to write a 100-word essay that evaluates all the gene candidates in the prompt. In the second step, a summarizer agent summarizes the output essay and distinguishes whether there is at least one gene directly linked to the phenotypes by outputting “Yes” or “No”. The “Yes” or “No” output for each case is then used to classify the cases into specific (Yes) or nonspecific (No) groups for subsequent evaluation.

### 2.4 HPO phenotype classification

In this study, we adopt the HPO Phenotype Classification methodology, as detailed in the Cohort Analyzer tool publication (Rojano *et al.* 2021), to evaluate HPO term specificity regarding the HPO hierarchy termed the dataset specificity index (DsI). This metric assesses whether the HPO terms used to phenotype the patients are general (closer to the root node in the hierarchy) or specific (further from the root node). The DsI calculation considers the distribution of HPO terms across various levels of the HPO tree structure, penalizing general terms near the root while rewarding specific terms closer to the leaf nodes. For classification analyses, we randomly sampled 90 cases each from BG, UDN, and DDD for 270 total patient cases.

### 2.5 Divide-and-conquer strategy

LLMs struggle to effectively address problems involving numerous options (Kafkas *et al.* 2023). To address this limitation, we used a divide-and-conquer strategy that consists of three steps: First, all gene candidates are randomly split into groups of five; second, LLMs estimate the probability of the gene candidates to be disease-causal within each group as their in-group probabilities; third, the final score for each gene is derived from the average of its in-group probability (Supplementary Figs S8 and S11, available as supplementary data at *Bioinformatics Advances* online). To formulate this pipeline, we give the final score *S(g_j_)* for a gene *g_j_* in the following equation:


$$S(g_{j})=\frac{1}{N}\sum_{i=1}^{N}Pr(g_{j})$$

Here, *N* is the total sampling number and *Pr(g_j_)* denotes the in-group probability of the gene *(g_j_)* provided by the LLMs. We divided the 25 gene candidates into five groups and the 50 gene candidates into 10 groups so that each group consists of 5 genes.

To demonstrate the utility of this strategy, we selected GPT-3.5 as the LLM for evaluation. Although GPT-4 was the top performer in our benchmark, we chose not to use it for this evaluation due to the significantly higher costs for input and output tokens via the OpenAI API.

### 2.6 Ranking strategy

We employ different strategies for interacting with proprietary and open-source LLMs due to their distinct capabilities. Specifically, GPT-3.5 and GPT-4 rank genes within their textual outputs. We follow the LM-Eval ranking strategy (Lin and Chen 2023) for open-source LLMs like Llama-2 and Mixtral-8x7B due to their ability to rank based on the probability of their output tokens. The LM-Eval strategy allows for ranking based on the probability of output tokens, providing a more efficient and interpretable method for evaluating the model’s response quality. These different ranking methods between the proprietary and open-source models cannot be unified because token probabilities are solely available for open-source models. This necessitates the use of a different prompt for open-source LLMs (Supplementary Fig. S2, available as supplementary data at *Bioinformatics Advances* online).

The open-source LLMs output either “Yes” or “No” to indicate the gene’s *g_j_* causality to the phenotypes. We estimate a log-likelihood ratio of “Yes” and “No” (Murphy 2012) for determining the genes’ causality to the phenotypes. The log-likelihood ratio is given as follows:


$$S(g_{j})=\log(\frac{Pr(\mathrm{Yes})}{Pr(No)})$$

where *Pr(Yes)* and *Pr(No)* denote the output probability of the “Yes” and “No” tokens. The ranking of genes is determined based on their respective log-likelihood values.

## 3 Results

### 3.1 Benchmark of large language model

**Figure 1.**
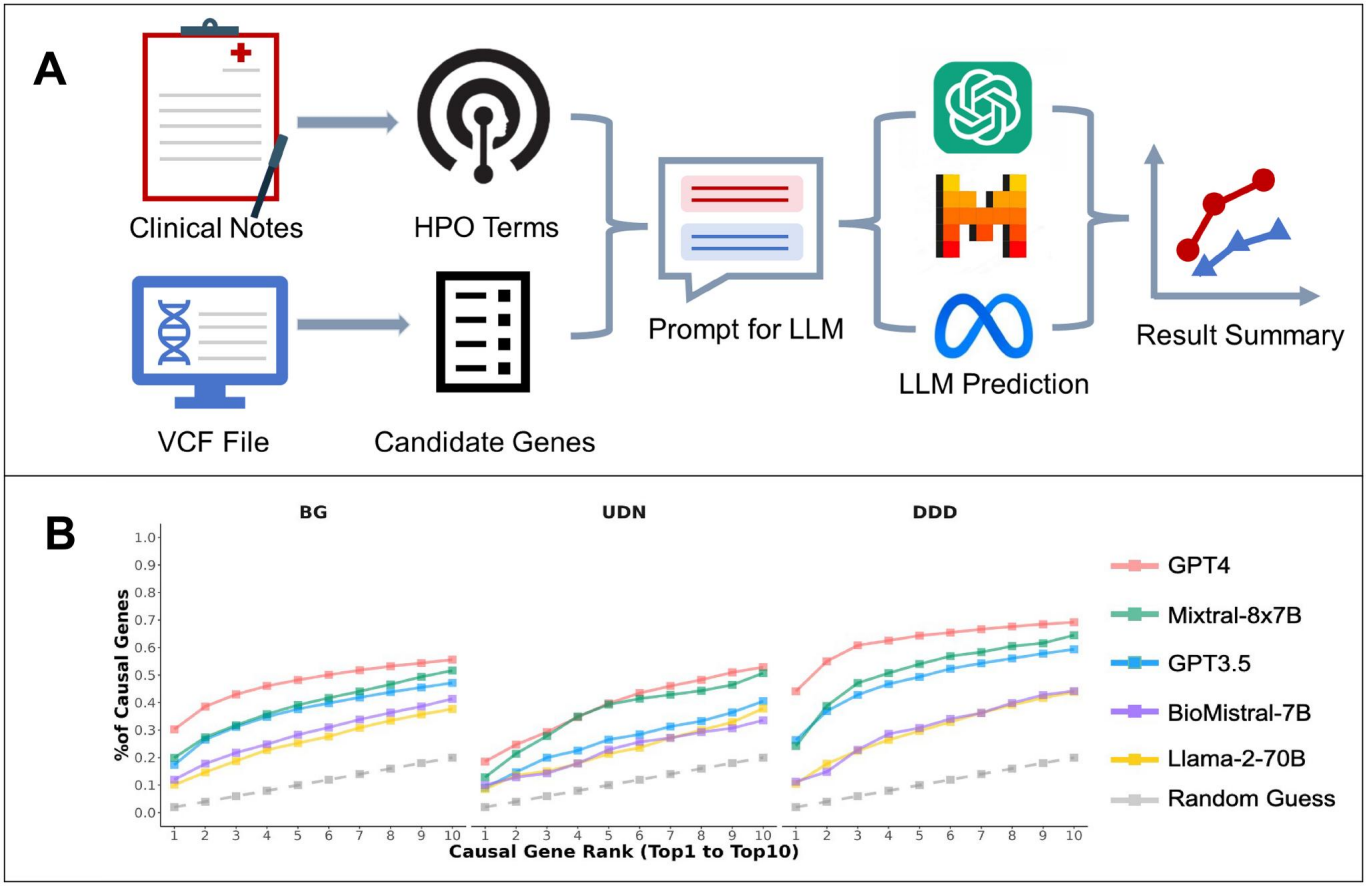
(A) The workflow for prompt creation and baseline comparison of LLMs. (B) Comparison of LLMs performance in ranking 50 causal genes including the likelihood a random guess selects the correct causal gene. The *x*-axis represents the rank threshold, from 1 to 10, at or within which the presence of causal genes is evaluated. The *y*-axis shows the proportion of total causal genes captured at each rank threshold, calculated as the cumulative number of causal genes at or within a given rank across all patient cases, divided by the total number of causal genes. Higher values at lower rank thresholds indicate better performance in prioritizing causal genes. GPT-4 demonstrates the highest proportion of causal genes at the top ranks, followed by Mixtral-8x7B, GPT-3.5, BioMistral-7B, and Llama-2-7B.

We began our study by conducting a baseline evaluation on several LLMs as applied to three datasets of patients with rare diseases from Baylor Genetics, Deciphering Developmental Disorders, and the Undiagnosed Diseases Network. The LLMs we examined included GPT-4 (OpenAI *et al.* 2023), GPT-3.5 (OpenAI *et al.* 2023), Mixtral-8x7B (Jiang *et al.* 2024), Llama-2-70B (Touvron *et al.* 2023), and a specialist biomedical model called BioMistral (Labrak *et al.* 2024). These represent proprietary models, open-source models, a mixture of experts model, and a domain specialist model. For each patient case, the LLM evaluated lists of candidate genes with lengths of 5, 25, and 50, along with the patient’s phenotypes, to observe its performance with different input sizes. The output was a ranked list of the genes with probabilities reflecting their likelihood of being associated with the phenotypes (Fig. 1A).

Employing an ensemble prompting strategy (Nori *et al.* 2023), which averaged the results of five iterations for each prompt, we obtained a consensus result for each LLM. As is common practice, we also employed mini-batching and output clipping to enhance processing speeds for larger open-source models. GPT-4 emerged as the standout performer, demonstrating superior performance to the other LLMs in correctly ranking causal genes as the most likely candidates (ranked first) for the patient’s disorder, highlighting its strong potential for use in genetic diagnostics (Fig. 1B, Supplementary Fig. S4, available as supplementary data at *Bioinformatics Advances* online). We additionally evaluated the open-source models verbally to illustrate the utility of using the ranking strategy based on using output token probabilities (Supplementary Fig. S5, available as supplementary data at *Bioinformatics Advances* online). Verbal evaluation showed decreased performance across the open-source LLMs, particularly in the case of Biomistral-7B and Llama-2-70B whose performance measured below that of randomly guessing the correct causal gene. This can likely be attributed to the number of outputs missing solutions or responses containing a refusal to attempt the ranking task.

The performance gap between GPT-4 and the next best model, Mixtral-8x7B, is most pronounced in the DDD dataset, where GPT-4 captures 40% of causal genes at rank 1, compared to 20% for both GPT-3.5 and Mixtral-8x7B (Fig. 1B, Supplementary Fig. S4, available as supplementary data at *Bioinformatics Advances* online). Across all datasets, Biomistral-7B and Llama-2-70B consistently exhibit low performance. Notably, identifying causal genetic genes proved more challenging for LLMs in the UDN dataset than in datasets from BG and DDD; this is likely because UDN patients tend to have had extensive prior screening without successful diagnosis or disorders not yet characterized, resulting in a wide range of phenotypes (Ramoni *et al.* 2017). DDD cases target congenital or early-onset severe phenotypes in children, optimizing the identification of a highly penetrant monogenic cause (Wright *et al.* 2015). BG consists of patients from a clinical diagnostic lab with varied cases, phenotypes, and genetic diagnoses (Mao *et al.* 2024). This stratification of patient case complexities explains why GPT-4 performs best on the DDD dataset.

### 3.2 Showcasing LLM performance across varied patient scenarios: insights on optimal conditions for high accuracy

**Figure 2.**
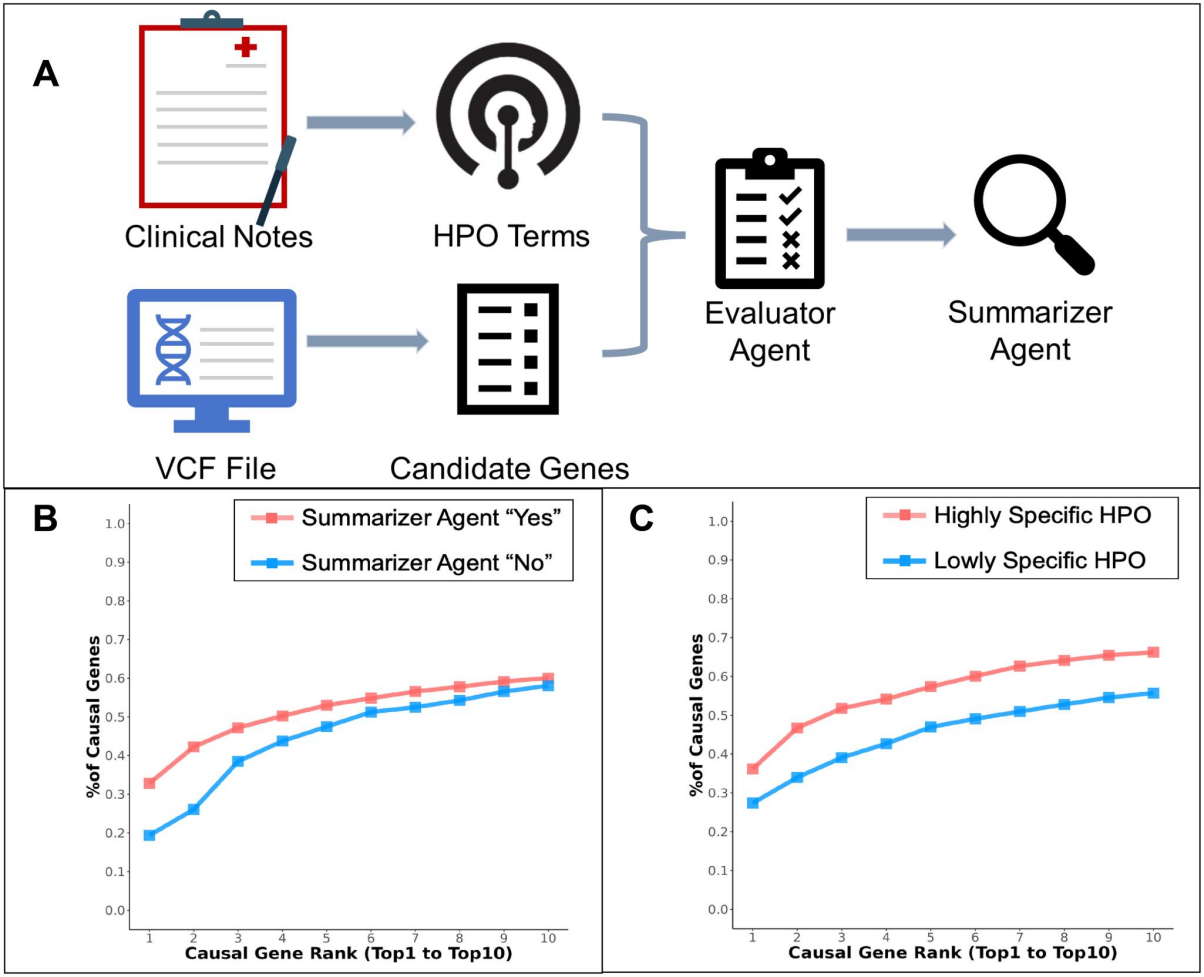
(A) A schematic depicting the multi-agent approach utilizing GPT-4, including prompt creation and subsequent processing done first by the evaluator agent and subsequently summarized by the summarizer agent. (B and C) Line graphs depicting the proportion of causal genes identified by GPT-4 from ranks 1 to 10, grouped by the presence of a clear association as determined by multi-agent approach and the HPO classification approach respectively, across all datasets.

Given the observed variability in patient presentations and datasets during the data preparation of the benchmark process, we classified the patient cases by phenotype and gene–phenotype association specificity. Given the observed variability in patient presentations and datasets during the benchmarking process, we categorized the patient cases based on phenotype and the specificity of gene–phenotype associations. This classification reflects the varying degrees of patient solvability, which can be directly related to the accuracy of predictions made by the LLM. For example, by classifying patients into “easy” and “difficult” solvability categories, clinical geneticists can better assess the reliability of LLM-generated predictions. When a patient case is classified as “easy,” the user can have greater confidence in the accuracy of the ranked candidate gene results. Thus building on this reasoning, we evaluated two approaches to accomplish this. We first developed a multi-agent system in which an evaluator agent is instructed to write a 100-word essay assessing gene–phenotype associations, then a summarizer agent analyzes the essay to classify cases as having the direct presence of gene–phenotype connections (Fig. 2A). We next evaluated an HPO Phenotype Classification methodology, which calculates the dataset specificity index (DsI) from the HPO-term distribution across the HPO hierarchy, to reward specific terms from deeper levels of the tree and penalize general terms from levels near the root. We applied both methods to three randomly sampled sets of 90 patient cases (to maintain similar evaluation set sizes) from each of our three datasets. Together the multi-agent and HPO classification strategies allowed for a refined analysis of cases based on their phenotypic and genotypic characteristics.

After grouping cases using the multi-agent approach, across all sample set sizes (5, 25, and 50), a clear distinction emerged between cases with positive (“yes”) and negative (“no”) association types across the datasets (Supplementary Fig. S9, available as supplementary data at *Bioinformatics Advances* online). We observed no more than 75 “yes” linked cases in UDN cases and more than 80, sometimes approaching 90 in the other two datasets out of 90 cases each, further confirming that UDN cases are more complex than are cases from BG or DDD. Amongst combined results from all the datasets, there is a clear distinction between the ability of the LLM to identify causal genes between either group of patient cases (Fig. 2B). For instance, for the positive association cases, the LLM correctly identifies more than 30% of causal genes as the most likely gene for causing the disease but only 20% for the negative association cases.

The HPO classification method effectively differentiated the datasets into “Highly Specific HPO” and “Lowly Specific HPO” categories based on the specificity of the phenotype terms used for the patient (Fig. 2C, Supplementary Fig. S8, available as supplementary data at *Bioinformatics Advances* online). The “Highly Specific HPO” cases consistently ranked higher in terms of causal gene rank across all three datasets (BG, UDN, and DDD), indicating that more specific phenotype descriptions lead to better performance in identifying the causal genes. Furthermore, the lower performance of the LLM on the UDN cases compared to the other datasets highlights the phenotypic and genotypic diversity of patients within each dataset and emphasizes the importance of considering these factors when evaluating LLMs for patient case analysis.

### 3.3 Literature and positional bias in LLMs

When considering the ranking abilities of LLMs, it is crucial to evaluate potential biases that may arise from positional biases within the prompt and the representation of genes in the literature. To investigate these factors, we conducted an analysis of LLM performance on the BG, UDN, and DDD datasets.

**Figure 3.**
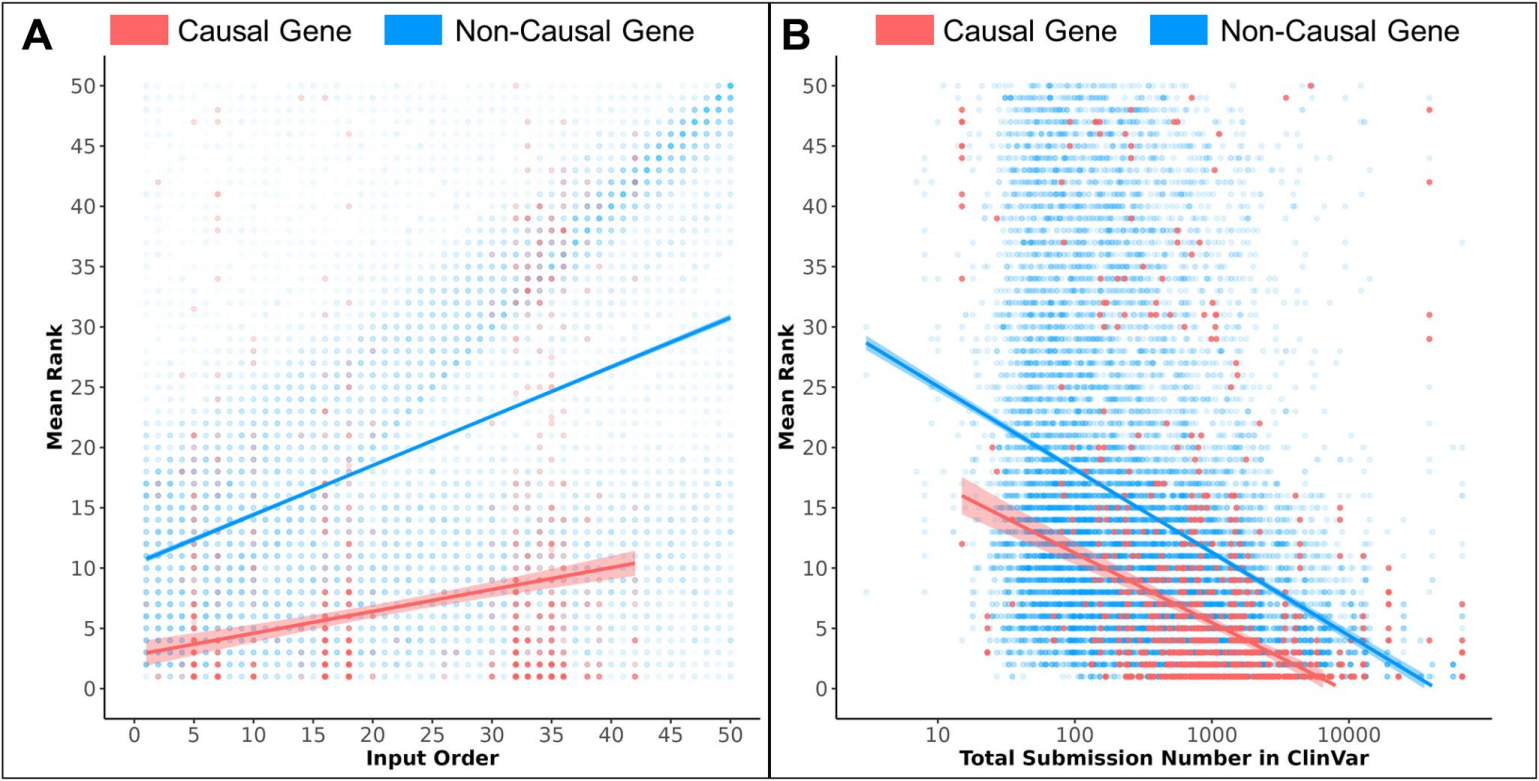
(A) A scatter plot showing a positive correlation between the order of the causal gene in the prompt and mean rank shown across 50 gene positions. (B) Another plot illustrates the inverse correlation between gene ranking and ClinVar submission numbers for BG, UDN, and DDD.

Our analysis revealed a distinct pattern in the relationship between the position of the true causal gene within the input prompt and its mean rank (Fig. 3A). As the positions of both causal genes and noncausal genes increased from 1 to 50, their mean rank also increased, indicating that the LLM assigns lower rankings to the gene when it appears later in the prompt. These observations are consistent with previous findings regarding positional bias (Ko *et al.* 2021, Zheng *et al.* 2023). Taken together, these results demonstrate that the gene ranking ability of an LLM is sensitive to the causal gene’s position within the input prompt. Later positions result in higher prioritization of the true causal gene on average, underscoring the importance of accounting for such biases.

We also identified a clear inverse correlation between the mean rank of a gene in these datasets and the ClinVar submission count that correspond to the gene (Fig. 3B). This finding suggests that LLMs may prioritize genes with greater representation in ClinVar, likely reflecting biases in both the literature and ClinVar itself, as well as the increased representation of these genes in the LLM pre-training data. It is established that genes that are more extensively studied tend to have higher submission numbers (Henrie *et al.* 2018, Magraner-Pardo *et al.* 2021), which could explain this observed trend.

### 3.4 Divide-and-conquer strategy addresses biases

To counteract previously mentioned biases, we introduce the divide-and-conquer strategy to handle various gene options and orders when assigning gene rankings. This three-step pipeline involves randomly splitting gene candidates into groups of five, estimating in-group probabilities using the GPT-3.5 model, and averaging these probabilities across sampling iterations to obtain final gene scores (Supplementary Fig. S10, available as supplementary data at *Bioinformatics Advances* online).

**Figure 4.**
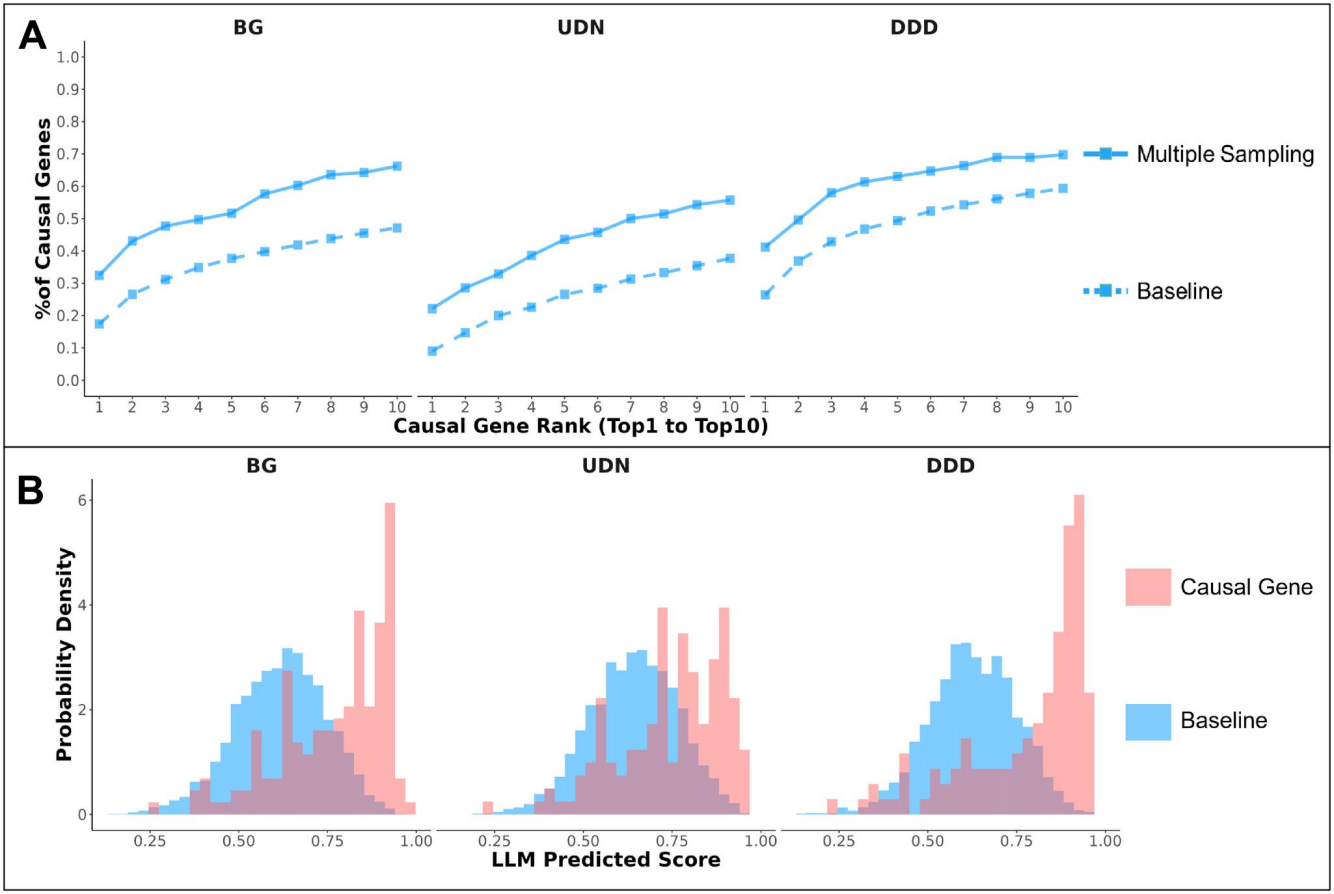
(A) Line graphs show the percentage of correctly identified causal genes from ranks 1 to 10 across BG, UDN, and DDD by GPT-3.5. Solid lines indicate divide-and-conquer strategy identification rates, with dashed lines marking baseline performance. (B) Histograms display the scoring distributions of causal and noncausal genes in each dataset, using the divide-and-conquer strategy.

The divide-and-conquer strategy effectively increases the performance of GPT-3.5 for each dataset across all ranks (Fig. 4A). We observed this strategy to be more effective when implemented on ranking longer candidate gene lists (Fig. 4A, Supplementary Fig. S12A, available as supplementary data at *Bioinformatics Advances* online). Causal genes consistently exhibit high scores when grouped with the remaining genes. Conversely, noncausal genes tend to display lower scores when grouped with causal genes, statistically scoring lower than causal genes (Fig. 4B). This strategy mitigates the literature and positional biases we have observed by employing uniform gene grouping and increasing sampling numbers. It also allows causal genes to exhibit high scores consistently while noncausal genes display lower scores when grouped with causal genes across variable lengths of candidate gene lists.

## 4 Discussion

Our study provides a comprehensive analysis of the ways in which Large Language Models (LLMs) can enhance gene-based prioritization, particularly in the context of rare genetic disorders. The performance benchmarks established for various LLMs, with GPT-4 as the frontrunner, underscore the potential advanced transformer architectures have to assist experts in clinical genomics. It is noteworthy that although we utilized token probabilities for open-source models, their performance still fell short of GPT-4’s enhanced capabilities despite GPT-4 using less effective verbal assessment. This potential is likely due to the extensive and varied training datasets that equip LLMs with an advanced understanding of both structured and unstructured medical data.

However, our findings also illuminate some challenges. Notably, certain cases were better addressed by LLMs than others, suggesting a variance in model efficacy that correlates with the complexity and specificity of the genetic and phenotypic data presented. This variability in performance emphasizes the need for models that can adapt to the high heterogeneity inherent in rare disease diagnosis.

A critical observation from our study is the apparent bias reflected in the existing literature and in genetic databases like ClinVar. Genes that are more frequently studied or reported tend to be prioritized by LLMs, potentially overshadowing less characterized but equally significant genes. This bias underscores a fundamental challenge in employing AI in medical diagnostics: the quality of AI outputs can only be as good as the data inputs.

To mitigate these biases and enhance gene-based prioritization’s reliability, we propose a divide-and-conquer strategy. By dividing the prediction into multiple prioritization processes and integrating the results, our approach not only diversifies the gene candidates considered but also reduces the influence of skewed data distributions. This strategy is crucial for advancing the utility of LLMs in clinical settings, where gene prioritization accuracy can directly influence patient outcomes.

In conclusion, although LLMs hold considerable promise to revolutionize genetic diagnostics, their implementation must be carefully managed to mitigate inherent biases and ensure equitable and comprehensive genetic analyses. Our study did not explicitly demonstrate examples of novel disease-gene associations discovered through LLM integration; however, we highlighted patient case conditions under which incorporating LLMs into gene prioritization workflows is likely to yield the most reliable downstream ranking of candidate causal genes for clinical interpretation. Future research should concentrate on refining these models to better address the complexity and heterogeneity of rare diseases, ultimately narrowing the gap between AI potential and real-world clinical application.

## Supplementary Material

vbaf148_Supplementary_Data

## Data Availability

The data underlying this article are available at: https://github.com/LiuzLab/GPT-Diagnosis
