# Supplementary material for: Survey and improvement strategies for gene prioritization with large language models
Source: Bioinform Adv. 2025 Jun 24;5(1):vbaf148. doi: 10.1093/bioadv/vbaf148 (PMC12263109; doi:10.1093/bioadv/vbaf148)
Supplement: vbaf148_Supplementary_Data [file vbaf148_supplementary_data.pdf]

Supplementary Materials

Output from Large Language Models

Take text directly from GPT models as output. From open-source models, we take the probability. For the GPT models, this value is not available. We can only get text from the GPT models.

We have different methods to interact with open-source LLMs such as Llama-2 and Mixtral compared with close-source LLMs such as ChatGPT. Open-source LLMs offer the probability of each output token, a feature unavailable in closed-source LLMs like ChatGPT. To illustrate, we utilize the following prompt when querying open-source LLMs. An example is provided below, where the placeholder [GENE NAME] represents the name of a gene.

### Given the following phenotypes in a patient: Global developmental delay, Generalized hypotonia, Failure to thrive, Tetralogy of Fallot, Hypertrophic cardiomyopathy, Abnormal facial shape. ### Are these phenotypes caused by a mutation in the [GENE NAME] gene? ### Use any available information including gene function, reports of genetic variants, expression sites, or animal model studies if direct human data is insufficient. ### Just answer Yes/No.

The LLM outputs either 'Yes' or 'No' to indicate the causality of a gene g_j to the phenotypes. We employ maximum likelihood estimation [3] to determine the causality of genes to the phenotypes. Specifically, the log-likelihood ratio of 'Yes' and 'No' indicates the log-likelihood value $S(g_j)$ that the gene g_j causes the phenotypes, as given in the following equation:

$$S(g_j) = log\left(\frac{Pr(Yes)}{Pr(No)}\right),$$

where $Pr(Yes)$ and $Pr(No)$ denote the output probability of the 'Yes' and 'No' tokens. The ranking of genes is determined based on their respective log-likelihood values.

Prompt Generation

For HPO data, we utilized the get_ontology() function from the ontologyIndex package to retrieve the HPO data. For gene data, we first extracted the gene symbols of causal variants from every patient case. Then, we used a sampling with pre-defined seeds to sample *n-k* gene symbols of non-causal variants from the filtered gene pool (*n* is the pre-defined number for all input genes and *k* is the number of gene symbols of causal variants of the given case). After the sampling, the causal genes and non-causal genes of every case will be combined in a shuffled order to reduce the positional bias. For investigating the effect of input order on the rank of causal genes, 5 different seeds were used in the shuffling process to get varied input orders.

GPT Query

Queries of GPT-3.5 and GPT-4 are performed by calling API. The model used for GPT-3.5 is gpt-3.5-turbo-1106. The model used for GPT-4 is gpt-4-1106-preview. The temperature is set to 1.0. For every patient case, 5 repeated queries with the same prompt will be performed for every case to evaluate the baseline robustness of the GPT models. Based on the output of API calling, another GPT-3.5 agent (gpt-3.5-turbo-1106, temperature = 1.0) will check and summarize the result into a list with the format format: Gene Name - Probability to reduce inconsistency of the output formats. Because of the consumption of

computational resources and the cost of API calls, we only did one query instead of 5 repeated queries for the multi-agent approach and correlation of input order and rank.

Result Summary

Based on the summarized output of LLMs, we matched the output gene names with the input gene names in the same case to assign ranks to every gene in the given case. Handling hallucinations, if the causal gene in the input list is not in the output list, the case will be abandoned for a further summary. Based on our observation, hallucinations with irrelevant outputs are rare. However, the output usually contains fewer genes compared with the input gene lists, especially when the input number of genes is 25 and 50. To measure the proportion of genes that can be matched during the result summary, we have causal_gene_output_ratio in the supplementary tables to evaluate the proportion of causal genes that can be matched in every batch of query and match_mean to evaluate the mean proportion of all input genes that can be matched in every batch of query. After assigning ranks to every gene, the metrics "% of causal genes" will be calculated by calculating the proportion of causal genes ranked ≤ n among all the causal genes in the batch of queries. Therefore, the abandoned cases due to missing causal genes will still be penalized as not within the given rank number.

Decreasing Inference/Generation Latency

To run larger open-source LLMs, we employ mini-batch inference and output clipping technologies. Specifically, mini-batch inference is a technique of feeding mini-batches of the input sequence to LLMs, thereby accelerating the overall generation process by distributing the inference processes. Output clipping is a technique utilized to reduce inference time by limiting the output token number, thereby shortening the output sequence. This approach is simply implemented by setting the `max_new_tokens` argument parameter to a low value. It proves particularly effective in tasks where concise answers are sufficient, such as confirming gene-disease associations with simple affirmative or negative responses. The reduction in output tokens can effectively reduce the latency without compromising performance.

Model	Gene Number	Dataset	Top1 Mean	Top1 SD	Top3 Mean	Top3 SD	Top5 Mean	Top5 SD	Top10 Mean	Top10 SD	Casual Gene Output Ratio	Total Gene Output Ratio
GPT3.5	5	BG	0.42	0.00	0.75	0.00	1.00	0.00	1.00	0.00	1.00	1.00
GPT3.5	5	UDN	0.40	0.03	0.75	0.01	0.99	0.01	0.99	0.01	0.99	1.00
GPT3.5	5	DDD	0.60	0.01	0.83	0.01	1.00	0.00	1.00	0.00	1.00	1.00
GPT3.5	25	BG	0.32	0.01	0.51	0.00	0.58	0.00	0.70	0.00	0.91	0.85
GPT3.5	25	UDN	0.25	0.02	0.42	0.02	0.51	0.01	0.66	0.02	0.87	0.82
GPT3.5	25	DDD	0.46	0.01	0.62	0.01	0.69	0.01	0.78	0.02	0.95	0.86
GPT3.5	50	BG	0.17	0.00	0.31	0.01	0.38	0.01	0.47	0.01	0.59	0.36
GPT3.5	50	UDN	0.09	0.01	0.20	0.02	0.27	0.02	0.38	0.05	0.54	0.39
GPT3.5	50	DDD	0.26	0.02	0.43	0.01	0.49	0.02	0.59	0.01	0.69	0.36
GPT4	5	BG	0.53	0.00	0.78	0.00	1.00	0.00	1.00	0.00	1.00	1.00
GPT4	5	UDN	0.55	0.01	0.77	0.01	1.00	0.00	1.00	0.00	1.00	1.00
GPT4	5	DDD	0.72	0.00	0.83	0.01	1.00	0.00	1.00	0.00	1.00	1.00
GPT4	25	BG	0.43	0.00	0.60	0.01	0.66	0.00	0.74	0.00	0.98	0.96
GPT4	25	UDN	0.32	0.01	0.52	0.01	0.62	0.02	0.75	0.03	0.94	0.96
GPT4	25	DDD	0.63	0.00	0.73	0.00	0.76	0.01	0.81	0.01	0.99	0.98
GPT4	50	BG	0.30	0.00	0.43	0.00	0.48	0.01	0.56	0.00	0.76	0.60
GPT4	50	UDN	0.19	0.01	0.29	0.04	0.40	0.02	0.53	0.01	0.67	0.52
GPT4	50	DDD	0.44	0.01	0.61	0.01	0.64	0.01	0.69	0.02	0.82	0.60

Supplementary Table S1: Benchmark results of the OpenAI models GPT-4 and GPT-3.5-turbo averaged across 5 repetitions of LLM interactions. The table includes the number of candidate genes to be ranked by the LLMs, the casual gene output ratio represents the average ratio of causal genes in the output to the total number of causal genes across all cases, representing the LLMs ability to include the causal gene in the ranking. The total gene output ratio is the proportion of genes in the input that are still represented in the output. The Top*N* columns represent the proportion of causal genes that were ranked at or within the ranking position *N*.

Model	Sample Size	Dataset	Top1 Mean	Top3 Mean	Top5 Mean	Top10 Mean
Llama-2-70B	5.00	BG	0.32	0.67	1.00	1.00
Llama-2-70B	5.00	UDN	0.34	0.70	1.00	1.00
Llama-2-70B	5.00	DDD	0.42	0.72	1.00	1.00
Llama-2-70B	25.00	BG	0.15	0.29	0.39	0.56
Llama-2-70B	25.00	UDN	0.14	0.24	0.38	0.57
Llama-2-70B	25.00	DDD	0.20	0.32	0.44	0.61
Llama-2-70B	50.00	BG	0.10	0.19	0.25	0.38
Llama-2-70B	50.00	UDN	0.09	0.15	0.21	0.38
Llama-2-70B	50.00	DDD	0.11	0.22	0.30	0.44
Mixtral-8x7B	5.00	BG	0.44	0.76	1.00	1.00
Mixtral-8x7B	5.00	UDN	0.44	0.74	1.00	1.00
Mixtral-8x7B	5.00	DDD	0.58	0.81	1.00	1.00
Mixtral-8x7B	25.00	BG	0.25	0.41	0.50	0.65
Mixtral-8x7B	25.00	UDN	0.19	0.38	0.51	0.69
Mixtral-8x7B	25.00	DDD	0.36	0.57	0.63	0.75
Mixtral-8x7B	50.00	BG	0.20	0.32	0.39	0.52
Mixtral-8x7B	50.00	UDN	0.13	0.28	0.39	0.51
Mixtral-8x7B	50.00	DDD	0.24	0.47	0.54	0.64
BioMistral-7B	5.00	BG	0.35	0.72	1.00	1.00
BioMistral-7B	5.00	UDN	0.33	0.71	1.00	1.00
BioMistral-7B	5.00	DDD	0.45	0.80	1.00	1.00
BioMistral-7B	25.00	BG	0.17	0.32	0.41	0.60
BioMistral-7B	25.00	UDN	0.15	0.30	0.35	0.60
BioMistral-7B	25.00	DDD	0.18	0.35	0.47	0.64
BioMistral-7B	50.00	BG	0.12	0.22	0.28	0.41
BioMistral-7B	50.00	UDN	0.10	0.14	0.23	0.34
BioMistral-7B	50.00	DDD	0.11	0.23	0.31	0.44

Supplementary Table S2: Benchmark results of the open-source LLMs. The table includes the number of candidate genes to be ranked by the LLMs. The Top*N* columns represent the proportion of causal genes that were ranked at or within the ranking position *N*.

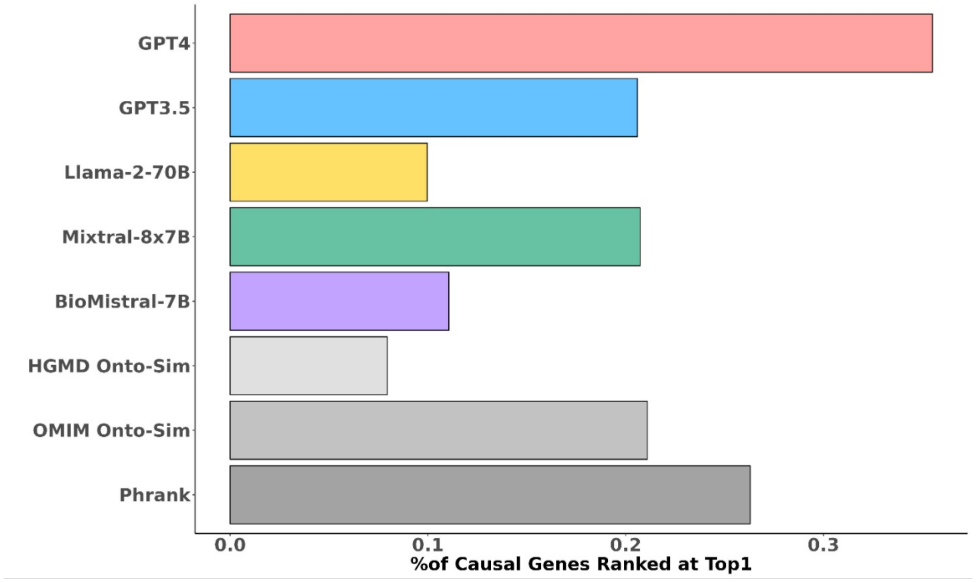

Supplementary Figure S1: Comparison of the performance of various traditional models and OpenAI models in ranking causal genes at the top position, measured as the percentage of causal genes ranked first across a combined benchmark dataset. This specifically measured phenotype-genotype relationships. The figure shows that GPT-4 outperforms all other models, including traditional tools such as Phrank, Onto-Sim and state-of-the-art variant prioritization methods.

A

Given the following phenotypes in a patient:
Duplicated collecting system
Global developmental delay
Generalized hypotonia
Failure to thrive
Bicuspid aortic valve
Abnormal facial shape

And considering these genes:
CASD1
NEO1
PASD1
ZNF560
KMT2D

Task: Rank the genes by their likelihood of causing the patient's phenotypes. Use any available information including gene function, reports of genetic variants, expression sites, or animal model studies if direct human data is insufficient.

Format: Provide a ranked probability (0.00 to 0.99) list with two columns (the name of the gene and the probability) without any introductory sentences and without any explanations. The format of every row of the ranked list should be: Gene Name – Probability.

B

Given the following phenotypes in a patient:
Given the following phenotypes in a patient: Global developmental delay, Generalized hypotonia, Failure to thrive, Tetralogy of Fallot, Hypertrophic cardiomyopathy, Abnormal facial shape. ### Are these phenotypes caused by a mutation in the [GENE NAME] gene? ### Use any available information including gene function, reports of genetic variants, expression sites, or animal model studies if direct human data is insufficient. ### Just answer Yes/No.

Supplementary Figure S2: (Panel A) An example of the prompt for GPT 3.5 and GPT 4. Panel B) An example of the prompt for open-source LLMs.

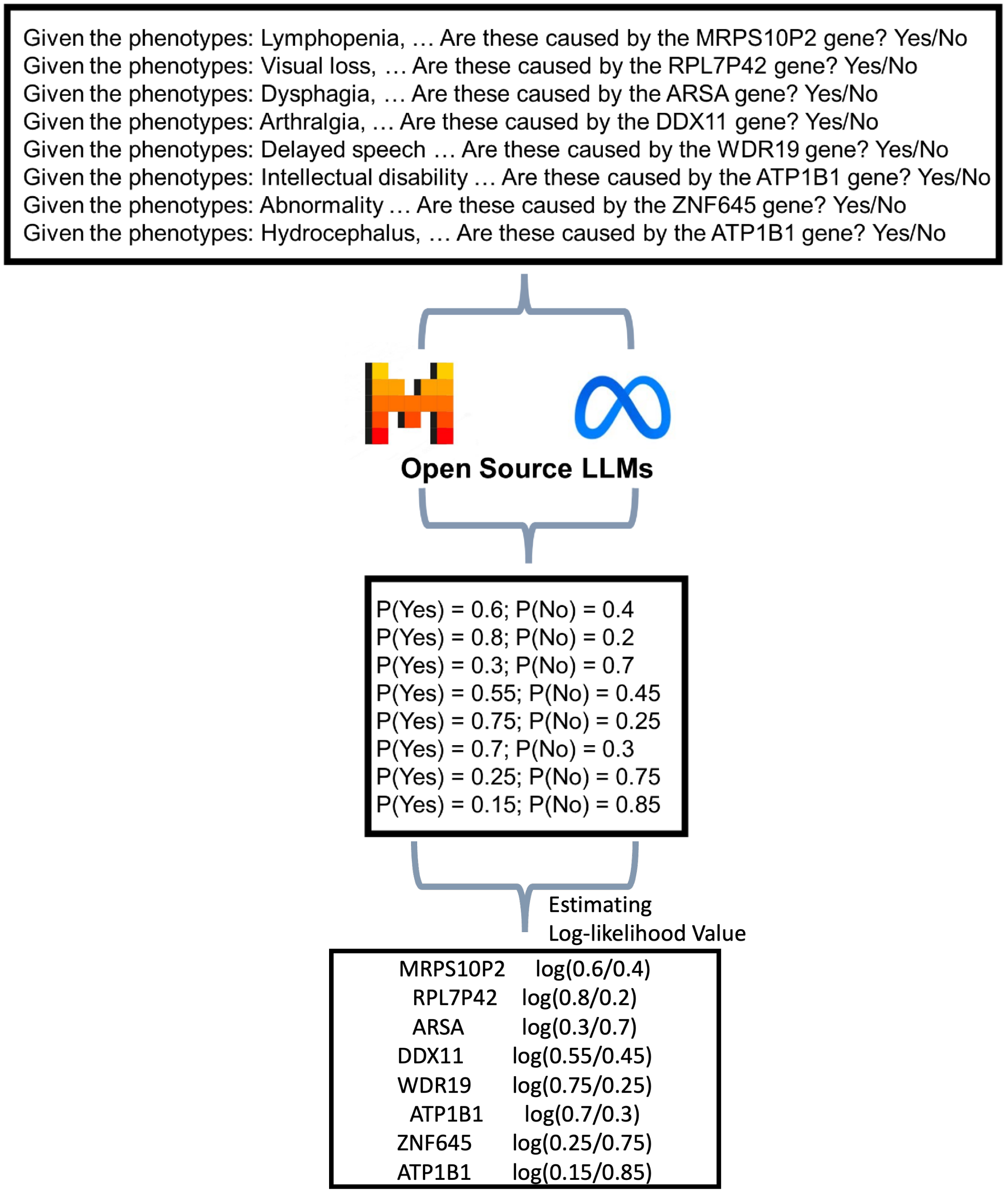

Supplementary Figure S3: An example of using mini-batch inference and log-likelihood estimation in open-source LLMs.

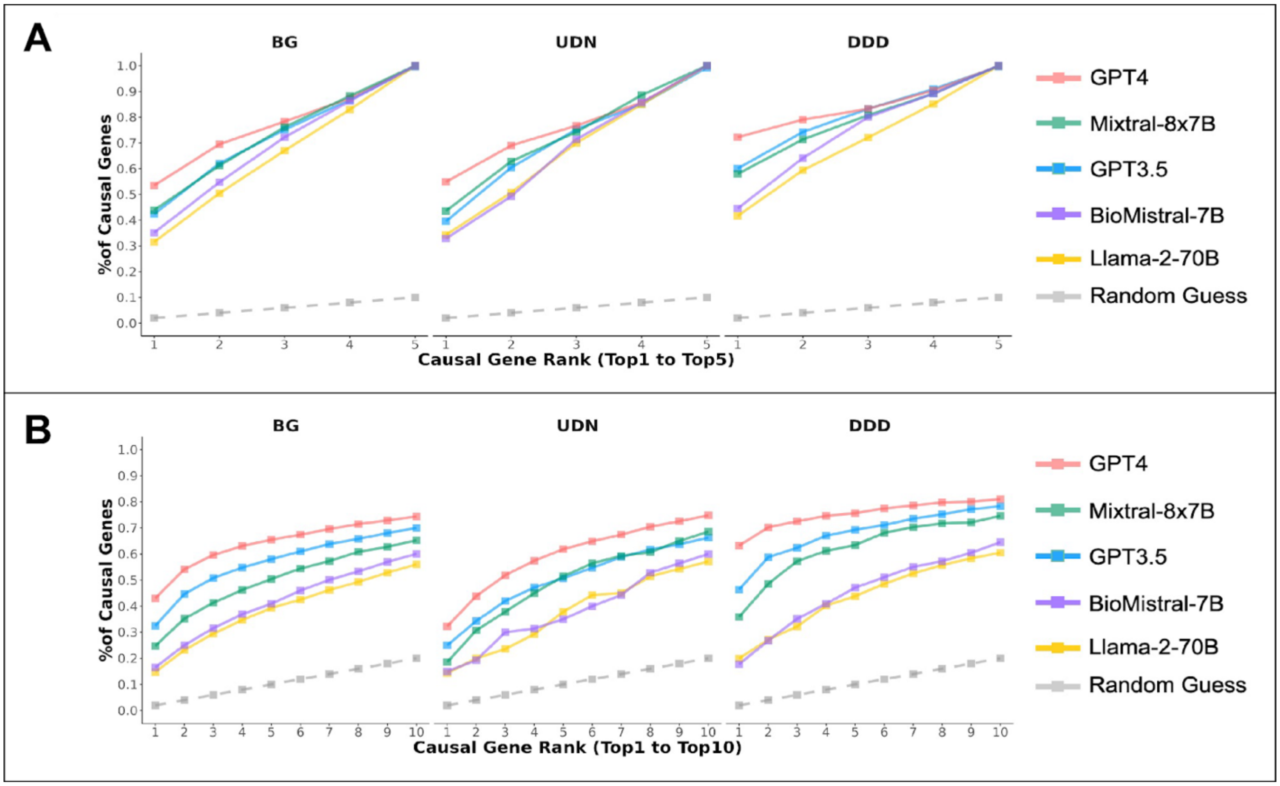

Supplementary Figure S4: (Panel A) Comparison of LLMs performance in ranking causal genes with 5 input genes per case. (Panel B) Comparison of LLMs performance in ranking causal genes with 25 input genes per case. Both panels include the likelihood of random guesses determining the correct causal gene.

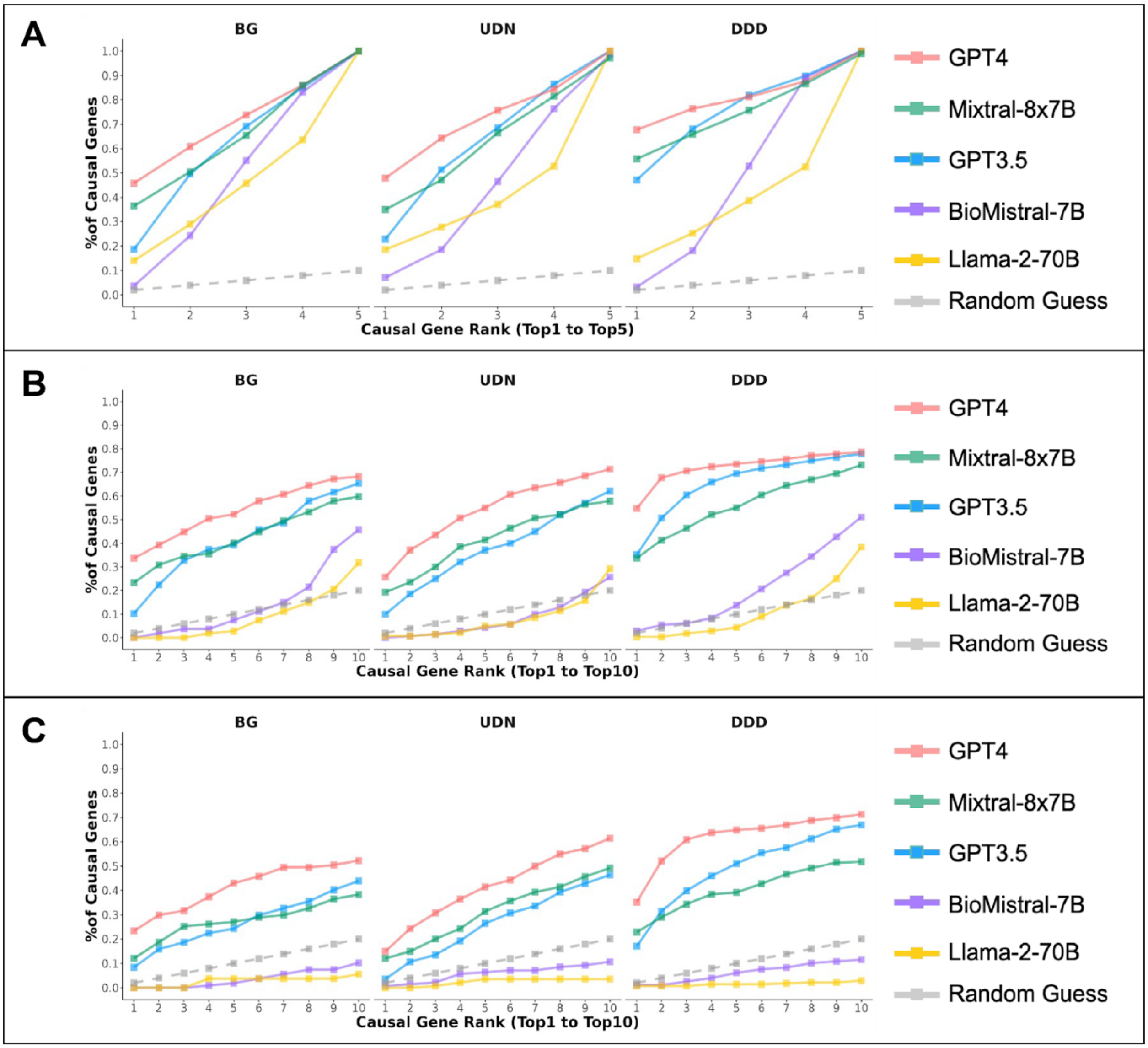

Supplementary Figure S5: Comparison with verbal assessment of open source LLMs as opposed to ranking via the probabilities of output tokens. Comparison of LLM performance in ranking causal genes across different input gene quantities (Panel A: 5 genes per case, Panel B: 25 genes per case, and Panel C: 50 genes per case), including random guess likelihood benchmarks.

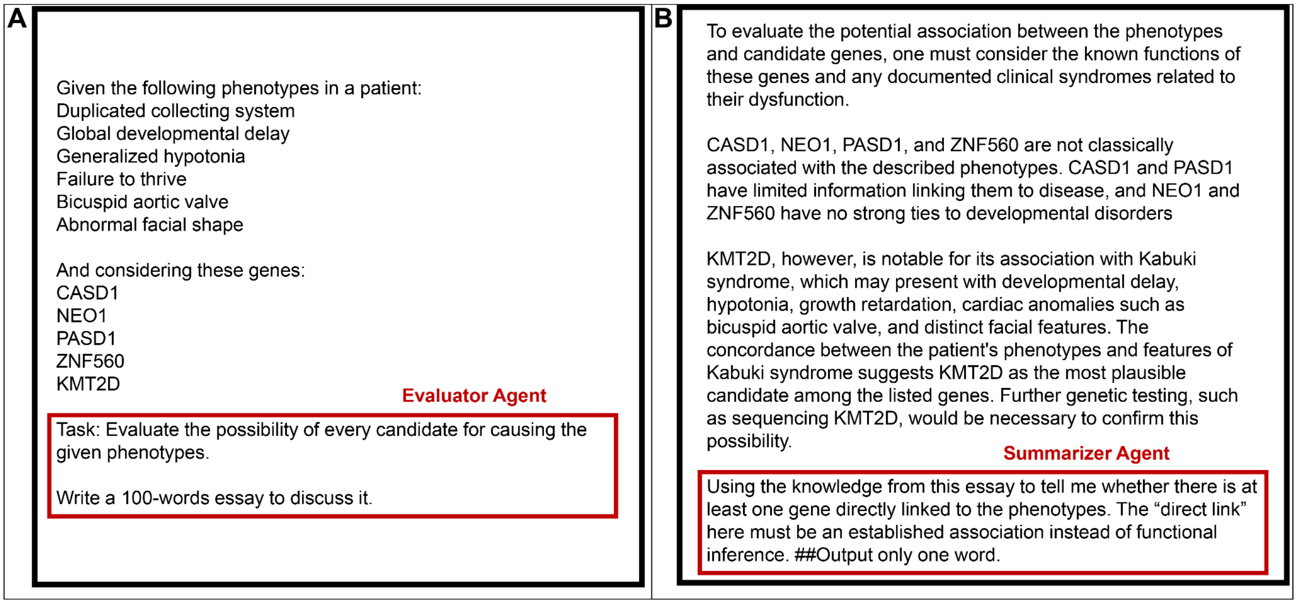

Supplementary Figure S6: (Panel A) An example of the prompt for the Evaluator Agent. (Panel B) An example of the prompt for the Summarizer Agent.

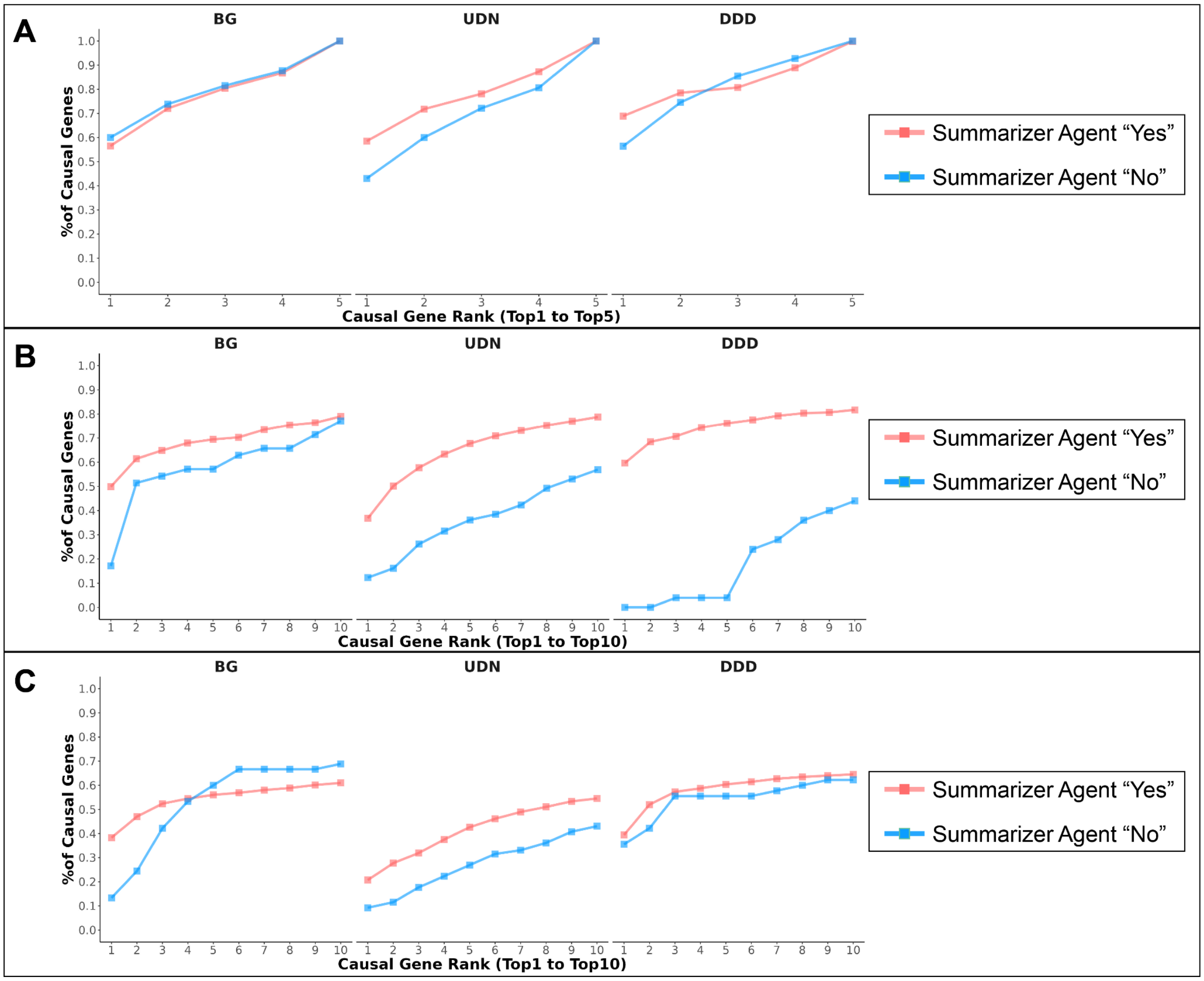

Supplementary Figure S7: (Panel A) Line graphs depicting the proportion of causal genes identified from ranks 1 to 10 under the multi-agent approach with 5 input genes per case. (Panel B) Line graphs depicting the proportion of causal genes identified from ranks 1 to 10 under the multi-agent approach with 25 input genes per case. (Panel C) Line graphs depicting the proportion of causal genes identified from ranks 1 to 10 under the multi-agent approach with 50 input genes per case.

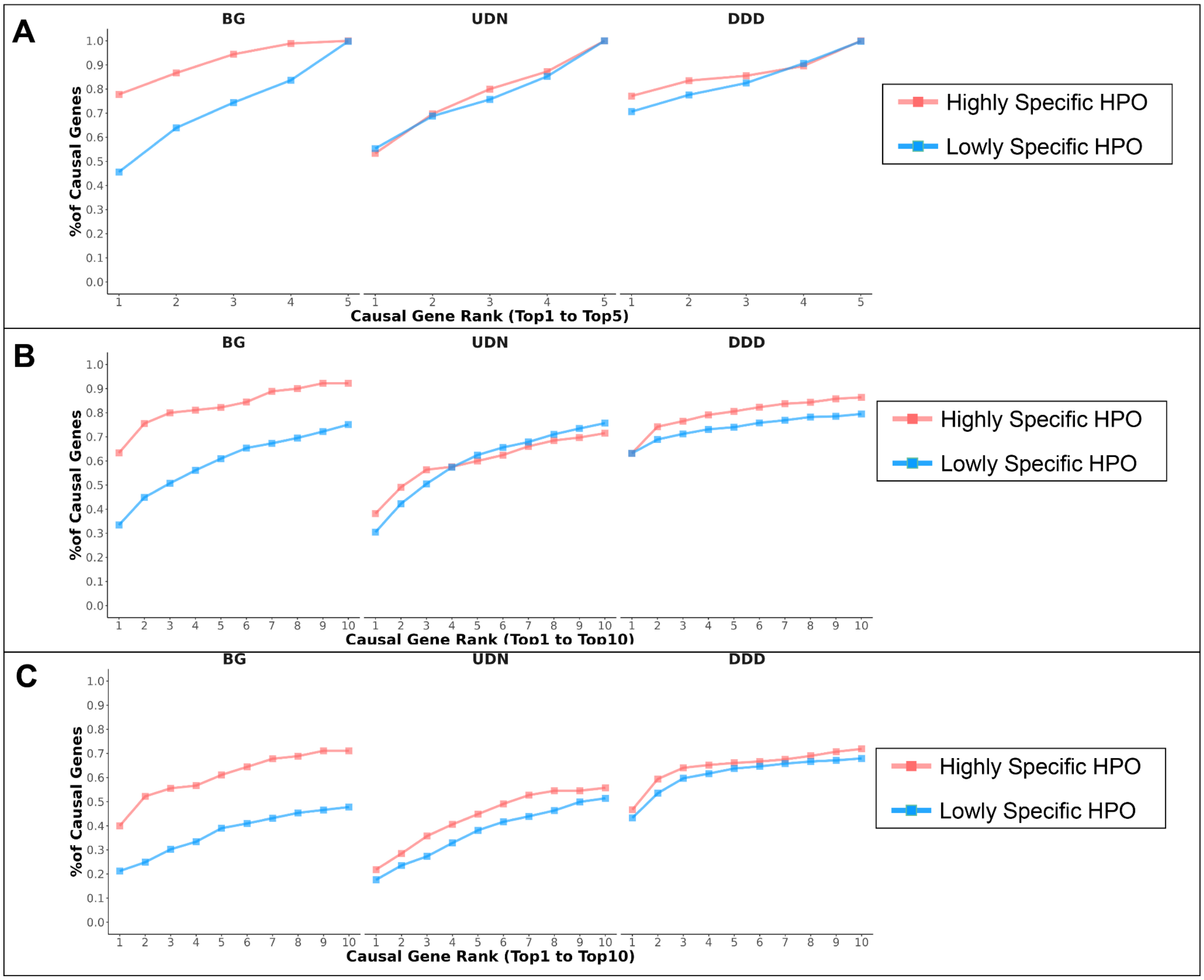

Supplementary Figure S8: (Panel A) Line graphs depicting the proportion of causal genes identified from ranks 1 to 10 under the HPO-classification approach with 5 input genes per case. (Panel B) Line graphs depicting the proportion of causal genes identified from ranks 1 to 10 under the HPO-classification approach with 25 input genes per case. (Panel C) Line graphs depicting the proportion of causal genes identified from ranks 1 to 10 under the HPO-classification approach with 50 input genes per case.

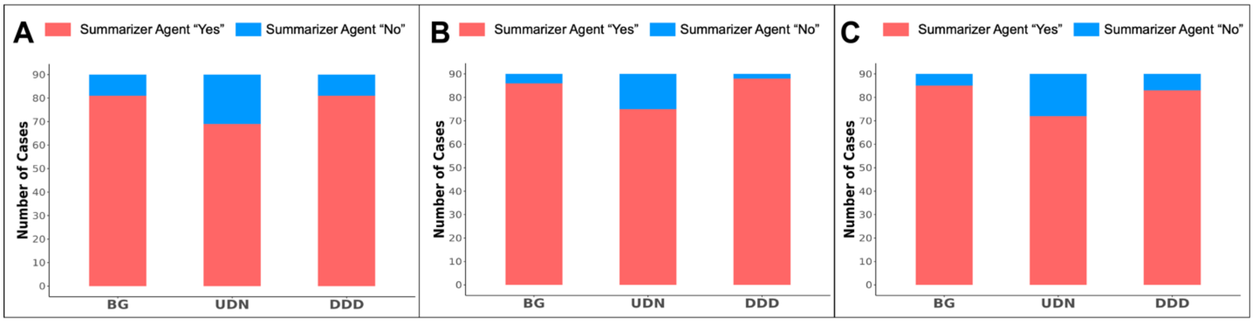

Supplementary Figure S9: From the left to right, plotted are the proportions of "Yes" and "No" cases using our multi-agent approach for sample sizes 5, 25 and 50.

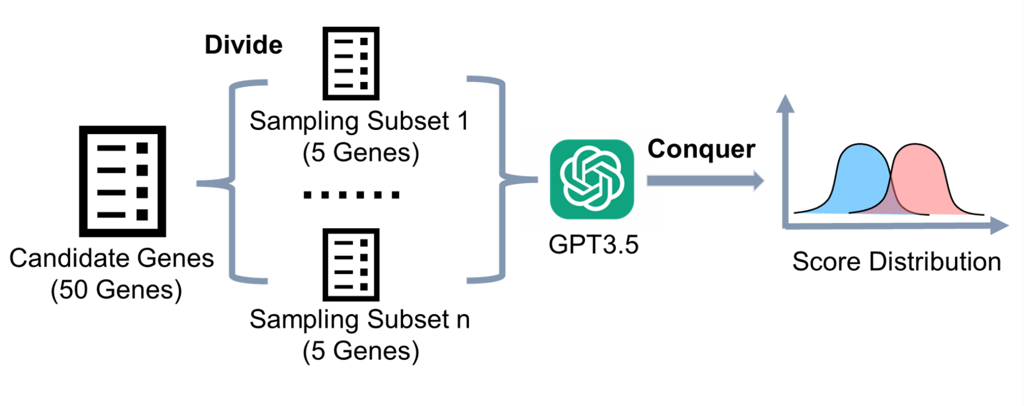

Supplementary Figure S10: The workflow implementing the Divide-Conquer Approach

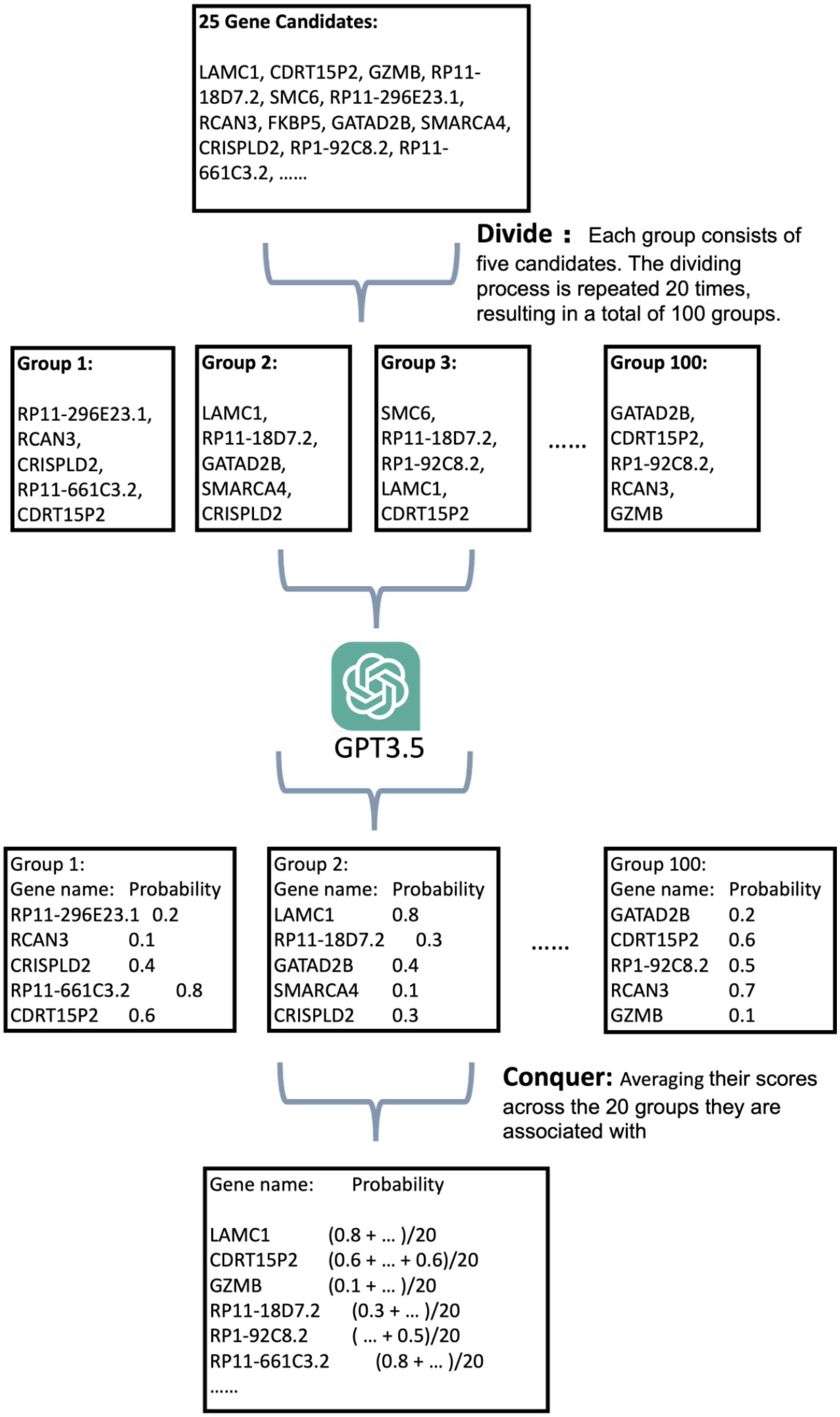

Supplementary Figure S11: An example of the Divide-and-conquer Approach. Let's consider a scenario where we have 25 gene candidates that need to be ranked. We begin by dividing these candidates into

groups, each consisting of five candidates. This process is repeated 20 times, resulting in a total of 100 groups. Consequently, every gene is part of 20 distinct groups.
Subsequently, we employ Figure S2 to estimate the ranking of genes within each group. Finally, we compute the average of their scores across the 20 groups they are associated with.

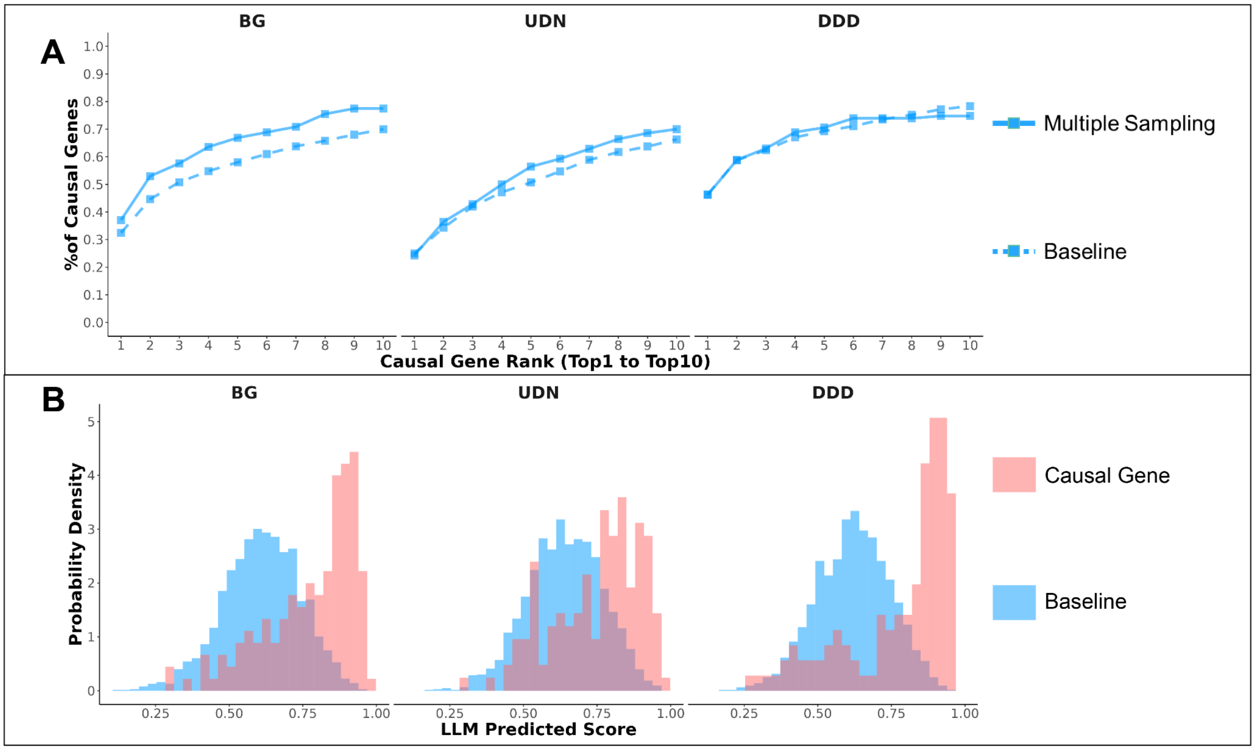

Supplementary Figure S12: (Panel A) Line graphs show the percentage of correctly identified causal genes from ranks 1 to 10 across BG, UDN, and DDD by GPT-3.5. Solid lines indicate divide-and-conquer strategy identification rates with 25 input genes per case, with dashed lines marking baseline performance. (Panel B) Histograms display the scoring distribution of causal (red) and non-causal (blue) genes in each dataset, using the divide-and-conquer strategy with 25 input genes per case.

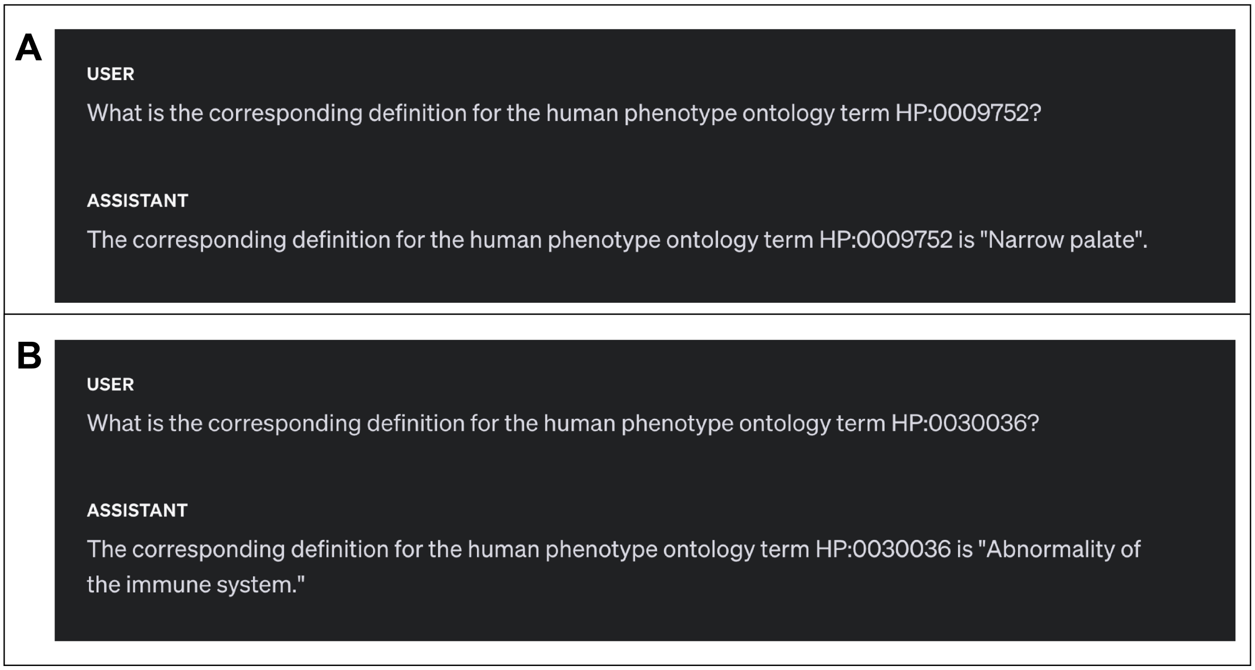

Supplementary Figure S13: (A) An interaction with GPT-3.5-turbo in the OpenAI playground with default settings beside temperature set to 0.5. Queried on the HPO ID HP:0009752 where the correct definition is “Cleft in skull base” the LLM returned a hallucination which we have observed is common behavior for LLMs in regards to understanding HPO IDs. (B) Another hallucination from the same LLM and parameters for the HPO term and ID “Isothenuria” HP:0030036.
